# Knockdown of long non‐coding RNA LINC00176 suppresses ovarian cancer progression by BCL3‐mediated down‐regulation of ceruloplasmin

**DOI:** 10.1111/jcmm.14701

**Published:** 2019-10-30

**Authors:** Lan Dai, Jixiang Niu, Yanli Feng

**Affiliations:** ^1^ Department of Gynaecology and Obstetrics Chinese Medicine Hospital in Linyi City Linyi China; ^2^ Department of General Surgery Chinese Medicine Hospital in Linyi City Linyi China

**Keywords:** B‐cell CLL/lymphoma 3, ceruloplasmin, epithelial‐mesenchymal transition, LINC00176, ovarian cancer

## Abstract

Ovarian cancer is a common malignancy among women with some clinically approved diagnostic coding gene biomarkers. However, long non‐coding RNAs (lncRNAs) have been indicated to play an important role in controlling tumorigenesis of ovarian cancer. Hereby, the aim of the study was to uncover the function of lncRNA LINC00176 in the development and progression of ovarian cancer by regulating ceruloplasmin (CP). Bioinformatics prediction in combination with RT‐qPCR analysis for the expression pattern of LINC00176 revealed that LINC00176 was highly expressed in ovarian cancer tissues as well as in ovarian cancer cell lines, respectively. LINC00176 was predominantly localized in the nucleus. Delivery of si‐LINC00176, oe‐LINC00176, si‐BCL3 and si‐CP plasmids was conducted to explore the effects of LINC00176 on ovarian cancer. Promoted proliferation, migration and invasion along with reduced apoptosis were observed in cells treated with oe‐LINC00176, while si‐BCL3 and si‐CP were able to block the promoting effects. Investigations with regard to the correlation between LINC00176 and promoter region of CP turned out to be positive *via* B‐cell CLL/lymphoma 3 (BCL3) by means of dual‐luciferase reporter gene assay, ChIP and RIP assays. Furthermore, oncogenic properties of the LINC00176/BCL3/CP axis were also demonstrated by tumour formation in vivo generated upon injecting cells in nude mice. Our results demonstrate that restored LINC00176 initiates tumorigenesis in ovarian cancer by increasing CP expression *via* recruiting BCL3, the mechanism of which represented a potential and promising therapeutic target for the disease.

## 1 INTRODUCTION

Ovarian cancer ranks as the 5th leading cause of cancer‐related mortality in the female population and characterized by histological and genetic tumours from epithelial, sex cord‐stromal and germ cells.^1^ As a global health issue, diagnosis of ovarian cancer usually is confirmed during a late stage and effective screening strategy at early stage has not been developed up to now.^2^ Besides, patients with ovarian cancer typically suffer from dismal prognosis while improvement of therapeutic approaches is still limited,^3^ which emphasizes on the urgency to discover novel remedies for ovarian cancer. Tumour cell metastasis is always evident in the late stage of ovarian cancer, where epithelial‐mesenchymal transition (EMT) has been identified to function as a critical regulator due to its promoting effects on cell dissemination and tissue invasion.^4^ Specifically, long non‐coding RNAs (lncRNAs) have emerged to be essential to EMT in gynaecologic cancers.^5^

LncRNAs, capable of regulating various pathological and physiological processes by functioning as oncogenes or antioncogenes, have been documented to function in different stages of ovarian cancer, thereby indicating their prognostic and therapeutic significance.^6^ LncRNAs have been confirmed to play an essential role in EMT in gynaecologic cancers.^5^ Specifically, overexpression of lncRNA DNM3OS, MEG3, and MIAT has been implicated in ovarian cancer.^7^ According to the bioinformatics results in our study, LINC00176 was revealed to be a differentially expressed lncRNA in ovarian cancer. LINC00176 is highly expressed in hepatocellular carcinoma while depleted expression LINC00176 can impede proliferation and induce necroptosis, highlighting that reduced expression of lncRNAs may be beneficial for cancer therapy.^8^ In addition, LINC00176 associates with the survival condition of patients suffering from oesophageal cancer.^9^ Based on the results from the LncMAP website, it was evident that LINC00176 could regulate the expression of ceruloplasmin (CP) *via* transcription factor B‐cell CLL/lymphoma 3 (BCL3). CP, consisting of 6 copper ions, is a 132‐kDa alpha2‐glycoprotein produced by the liver and secreted into the circulation.^10^ CP can function as a tumour progression marker of epithelial ovarian cancer due to its significantly higher expression in the ascitic fluids of patients with intrinsic chemoresistance.^11^ BCL3 has been acknowledged as another important proto‐oncogene in context of ovarian cancer that a high BCL3 expression has been evident in human ovarian cancer tissue where ovarian cancer cell survival, proliferation and migration are promoted, respectively.^12^ BCL3 involves in key oncogenic pathways in association with cell death and apoptosis in solid tumours and can inhibit ovarian cancer proliferation through interplay with microRNA‐125b (miR‐125b).^13^ However, the functioning of BCL3 cooperates with lncRNA with respect to regulation of cancer progression is unknown. Therefore, we proposed a hypothesis asserting that the LINC00176/BCL3/CP axis may play a novel role in the progression and development of ovarian cancer via confirmation by a series of in vitro and in vivo experiments.

## 2 MATERIALS AND METHODS

### 2.1 Ethics statement

The study was conducted under the approval of the Institutional Review Board of Chinese Medicine Hospital in Linyi City. All participants provided written informed consent prior to this study. The animal protocol and experiment procedure were conducted under the approval of the Institutional Animal Care and Use Committee of Chinese Medicine Hospital in Linyi City. Animals in our study were used for research purpose only.

### 2.2 Microarray‐based gene expression profiling

The ovarian cancer‐related gene expression data set http://www.ncbi.nlm.nih.gov/geo/query/acc.cgi?acc=GSE38666 was downloaded from the Gene Expression Omnibus (GEO) database (https://www.ncbi.nlm.nih.gov/geo/), followed by conducting a procedure of screening of differential genes using R language with |log2FC| > 1.5 and *P *Value < .05 as threshold. Heatmap depicting differential gene expression was established subsequently.

### 2.3 Sample collection and ovarian cancer cell lines

A total of 56 patients with ovarian cancer who had received partial excision from December 2014 to December 2016 after diagnosis in Chinese Medicine Hospital in Linyi City were enrolled and adjacent tissues resected and kept properly. The adjacent tissue pathologically diagnosed as lesion tissues without cancer cells was resected from upon operating the operation. The expression of LINC00176 in 5 ovarian cancer cell lines (CaoV‐3, 3AO, sk‐ov‐3, HO8910 and A2780) and normal ovarian epithelial cell line (CHO 1‐15) (subscript 500, ATCC^®^ CRL‐9606™) serving as control was determined by means of reverse transcription‐quantitative polymerase chain reaction (RT‐qPCR) procedure. The cell line exhibiting the highest LINC00176 expression was selected for further experimentation.

### 2.4 Cell treatment

Plasmids of si‐LINC00176, oe‐LINC00176, si‐BCL3, si‐CP and their negative controls (NCs) were transfected into HO8910 cells. The aforementioned target plasmids were purchased from Dharmacon, Lafayette, CO, USA. HO8910 cells were seeded in a 6‐well plate at a density of 3 × 10^5^ cells/well and then transfected using Lipofectamine 2000 (Invitrogen Inc) upon reaching the cell growth density of 80%. Then, 250 µL Opti‐MEM (Gibco Company) was added to dilute 4 µg target plasmids and 10 µL Lipofectamine 2000, respectively. After standing at room temperature for 5 minutes, the aforementioned components were mixed together and allowed to react for 20 minutes. Following a regimen of 6‐hours culture in an incubator at 37°C with 5% CO_2_, the medium was renewed for further culture. Finally, the cells were harvested 36‐48 hours after culturing.

### 2.5 RNA isolation and quantitation

Total RNA was extracted using the TRIzol reagent (15596026, Invitrogen). Then, the extracted RNA was reversely transcribed into complementary DNA (cDNA) in strict accordance with the provided instructions of the reverse transcription kit (RR047A, Takara). The SYBR Premix EX Taq kit (RR420A, Takara) was employed for sample loading. RT‐qPCR was performed on an ABI 7500 qPCR instrument (ABI Company) subsequently. Three duplicate wells were set for each sample. The aforementioned primers (Table 1) were synthesized by Shanghai Biotech Co., Ltd.. With regard to β‐actin as the internal control, the fold changes of gene expression were calculated on the basis of relative quantification (2^−ΔΔ^ *^C^* ^t^ method).

**Table 1:** Primer sequences for reverse transcription‐quantitative polymerase chain reaction

Gene	Primer sequences
LINC00176	F: 5′‐GGGTCCAGCTCAAATCGTTG‐3′
	R: 5′‐ATGTAAGVGVTCCGGAACTT‐3′
CP	F: 5′‐TCCGCAAATCCAGTTCCTCC‐3′
	R: 5′‐AAGTTTGGCAATGCGTGGAC‐3′
β‐actin	F: 5′‐CGCTTCGGCAGCACATATACTA‐3′
	R: 5′‐CGCTTCACGAATTTGCGTGTCA‐3′

Abbreviations: CP, ceruloplasmin; F, forward; R, reverse.

### 2.6 Fluorescence in situ hybridization (FISH)

FISH assay was conducted to identify the subcellular localization of LINC00176. The coverslip was placed in a 24‐well plate, where cells were seeded at a density of 6 × 10^4^ cells/well. When cell confluence reached about 85%, the coverslip was rinsed using phosphate‐buffered saline (PBS), fixed utilizing 1 mL 4% paraformaldehyde solution and treated with proteinase K (2 µg/mL), glycine and acetylation reagent, respectively. Then, the cells were pre‐hybridized with 250 µL LINC00176 probes (300 ng/mL) (Eurogentec, Seraing, Belgium) overnight. After 3 rinses using PBS with Tween‐20 (PBST), the nucleus was stained using PBST‐diluted 4′,6‐diamidino‐2‐phenylindole (DAPI) (1:800) in a 24‐well plate for 5 minutes. After that, the cells were sealed using fluorescence decay‐resistant medium. Five fields were randomly selected for microscopic observation and photography under a fluorescence microscope (Olympus Optical Co., Ltd).

### 2.7 RNA binding protein immunoprecipitation (RIP) assay

The BCL3‐induced enrichment of LINC00176 was determined using a RIP Kit (Millipore). After a pre‐cooled PBS rinse, the HO8910 cells were lysed with equal volume of lysis in ice bath for 5 minutes. The supernatant was attained through centrifugation at 21 912 × *g* and 4°C for 10 minutes. A portion of the cell extract was used as input, while the remaining was probed with the BCL3 antibody (ab27780, Abcam lnc) for coprecipitation. Immunoglobulin G (IgG) antibody (ab2410, Abcam Inc) served as NC. RNA was extracted from the sample and input after protease K detachment, followed by RT‐qPCR detection.

### 2.8 Chromatin immunoprecipitation (ChIP) assay

HO8910 cells were fixed using formaldehyde solution for 10 minutes to initiate DNA‐protein cross‐link. Chromatin fragments were obtained by application of ultrasonic sound (10 seconds each time at an interval of 10 seconds, 15 times in total). The supernatant was collected through centrifugation at 12 000 × *g* and 4°C for 10 minutes, subpacked into 2 tubes, and incubated with 2 µg NC antibody to IgG (ab2410, Abcam Inc) and 2 µg target protein‐specific antibody to BCL3 (ab27780, Abcam Inc) and p50 (sc‐166588, Santa Cruz Biotechnology, Inc) at 4°C overnight. The DNA‐protein complex was precipitated using protein agarose/Sepharose, followed by centrifugation at 16 099 × *g* for 5 minutes. The supernatant was discarded, and the non‐specific complex was washed. De‐cross‐linking was conducted at 65°C overnight. DNA fragment was purified and extracted using phenol/chloroform. The binding of BCL3 to the CP promoter was determined by RT‐qPCR using CP‐specific primer.

### 2.9 Co‐immunopreciptation (Co‐IP)

Cells in each group were lysed with lysis buffer containing 50 mmol/L Tris‐HCl (pH = 7.4), 150 mmol/L NaCl, 10% glycerol, 1 mmol/L ethylenediaminetetraacetic acid (EDTA), 0.5% NP‐40 and protease inhibitor mixture, and then centrifuged, followed by removal of cell fragments. Next, the removed cell lysate was incubated with 1 µg rabbit polyclonal antibody to BCL3 (ab27780, Abcam Inc) and 15 µg protein A/G beads (Santa Cruz Biotechnology, Inc) for 2 hours. After extensive washing, the beads were boiled for 5 minutes at 100°C. The protein was separated using sodium dodecyl sulphate‐polyacrylamide gel electrophoresis and transferred onto the nitrocellulose membrane (Merck Millipore), followed by application of Western blot analysis.

### 2.10 Dual‐luciferase reporter gene assay

According to the bioinformatics analysis, target genes of LINC00176 were predicted. The promoter region of CP was constructed onto the pGL3‐Basic vector (Promega Corp.) to produce pGL3‐CP recombinant vector. The correctly sequenced luciferase reporter plasmid was successfully acquired and cotransfected with oe‐LINC00176, oe‐NC, si‐LINC00176 and si‐NC into HEK293T cells, which were then collected and lysed 48 hours later. The luciferase activity was measured using a dual‐luciferase detection kit (K801‐200, Biovision Inc) in a dual‐luciferase reporter gene analysis system (Promega Corp.). Renilla luciferase served as an internal reference. The relative activity of luciferase was indicative of the activity degree of target reporter gene, which equalled the ratio of relative light unit (RLU) of firefly luciferase to RLU of Renilla luciferase.

### 2.11 5‐ethynyl‐2′‐deoxyuridine (EdU) staining

EdU solution (culture medium:EdU solution = 1000:1) was added into the plate for 2‐hours incubation at room temperature. The plate was then fixed using 4% paraformaldehyde solution for 30 minutes, incubated with glycine for 8 minutes and washed with PBS containing 0.5% Triton X‐100. The sample was then stained using Apollo^®^ Liquor for 30 minutes at room temperature under conditions devoid of light and rinsed using methanol and PBS. Hoechst 3334 solution was then added for another regimen of 20‐minutes incubation at room temperature in conditions devoid of light. The fluorescence microscope was employed for observation with 3 randomly selected fields of view (200×). Cell proliferation rate (%) = the number of proliferative cells (EdU‐stained cells)/the number of total cells (Hoechst 33342‐stained cells) × 100%.

### 2.12 Flow cytometry

HO8910 cells in the logarithmic growth phase were seeded in a 6‐well plate at a density of 2 × 10^5^ cells/well and collected 48 hours later. Subsequently, the single‐cell suspension was centrifuged at 4000 × *g* for 3 minutes with the supernatant aspirated, followed by the addition of 400 µL Annexin V/fluorescein isothiocyanate (FITC) solution. After incubation on ice for 15 minutes in conditions devoid of light exposure, the cells were stained with 10 µL propidium iodide (PI) liquor for 5 minutes under conditions void of light. Finally, flow cytometry was conducted for detection of cell apoptosis at an excitation wavelength of 488 nm.

### 2.13 Transwell migration assay

HO8910 cells in the logarithmic growth phase were subjected to starvation for 24 hours, detached, centrifuged and re‐suspended to assure the final concentration as 2 × 10^6^ cells/mL. Then, 0.2 mL serum‐free cell suspension was added into the apical chambers of Transwell while the pre‐cooled Dulbecco's modified Eagle's medium (DMEM) containing 20% foetal bovine serum (FBS) was added to the basolateral chambers. After a regimen of 24‐hours culture at 37°C under 5% CO_2_, Transwell chambers were removed and the cells in the apical chamber as well as inner side of basement membrane were wiped using wet cotton swabs. Next, the cells were then fixed in methanol for 30 minutes and stained using 0.1% crystal violet for 20 minutes at room temperature. At last, the migrating cells were counted with 100× magnification under an inverted microscope in 5 randomly selected visual fields.

### 2.14 Transwell invasion assay

The extracellular matrix (ECM) gel was placed at 4°C overnight and diluted to 1 mg/mL the next day with serum‐free medium at a ratio of 1:9. Subsequently, 40 µL ECM was added to the polycarbonate membrane on the apical chamber and subjected to incubation at 37°C with 5% CO_2_ for 5 hours until the ECM polymerized into gel. Next, 70 µL DMEM was added into each well, followed by incubation at 37°C for 0.5 hour to prepare a hydrated gel. Subsequently, HO8910 cells were subjected to starvation for 24 hours, detached, centrifuged and re‐suspended using FBS‐free DMEM until the final concentration was 2 × 10^6^ cells/mL. Then, 0.2 mL cell suspension was added into the apical chambers with hydrated basement membrane while 700 µL pre‐cooled DMEM containing 20% FBS was added to the basolateral chambers. After 24‐hours culture at 37°C under 5% CO_2_, Transwell chambers were removed and cells in the apical chamber and the inner side of basement membrane were wiped using wet cotton swabs. Then, the cells on the outside of the basement membrane were fixed using methanol for 30 minutes and stained with 0.1% crystal violet for 20 minutes. At last, the invasive cells were counted with 100× magnification under an inverted microscope from 5 randomly selected visual fields.

### 2.15 Western blot analysis

Total protein was extracted from the tissues or cells using radioimmunoprecipitation assay (RIPA) lysis containing phenylmethylsulfonyl fluoride (PMSF), with the concentration determined using a bicinchoninic acid (BCA) protein assay kit. Then, 50 µg protein was dissolved in 2× sodium dodecyl sulphate (SDS) buffer solution and boiled at 100°C for 5 minutes. The sample was then subjected to SDS‐polyacrylamide gel electrophoresis (SDS‐PAGE) and transferred onto a polyvinylidene fluoride (PVDF) membrane. Subsequently, the membrane was blocked utilizing 5% skim milk at room temperature for 1 hour and then incubated at 4°C overnight with the following primary antibodies purchased from Abcam Inc: rabbit monoclonal antibody to E‐Cadherin (1:1000, ab133597), rabbit polyclonal antibody to N‐cadherin (1:1000, ab18203), rabbit monoclonal antibody to vimentin (1:1000, ab92547), rabbit polyclonal antibody to CP (1:1000, ab48614), rabbit polyclonal antibody to Bcl3 (1:1000, ab27780) and rabbit monoclonal antibody to p50 (1:2000, ab32360). Meanwhile, the rabbit polyclonal antibody to glyceraldehyde‐3‐phosphate dehydrogenase (GAPDH) (1:1000, ab9485) was regarded as an internal reference. Next, the horseradish peroxidase (HRP)‐conjugated secondary antibody goat anti‐rabbit IgG (1:20 000, ab205718, Abcam Inc) was added to the membrane for incubation for 1 hour. The membrane was then developed using an enhanced chemiluminescence (ECL) kit (BB‐3501, Ameshame) and visualized under a gel imaging instrument. Photography was conducted using the Bio‐Rad image analysis system (Bio‐Rad). The grey value was quantified and analysed using the Quantity One v4.6.2 software. The level of GAPDH expression was regarded as the loading control.

### 2.16 Xenograft tumour in nude mice

A total of 36 BALB/c mice of either sex (age: 4 weeks; weight: 18‐25 g) were housed in a specific pathogen‐free (SPF)‐grade animal room. Cells (2 × 10^6^) transfected with oe‐LINC00176, si‐LINC00176 and si‐CP along with their corresponding NCs were re‐suspended in 50 µL normal saline, mixed with 50 µL Matrigel matrix and subcutaneously injected into the mice. The tumour volume was under observation after injection with long axis (A) and short axis (B) of tumour measured and recorded. The exact tumour volume was calculated on the basis of the following formula: V (mm^3^) = (A × B^2^)/2. Growth curve was displayed by plotting tumour volume against time. The tumour‐bearing mice were killed on the 4th week. The entire implant tumour was resected, weighed and photographed.

### 2.17 Statistical analysis

SPSS 21.0 software (IBM Corp.) was employed for data analysis. The experiment was repeated 3 times independently. The measurement data were expressed as mean ± standard deviation. Comparison of paired design between two groups following normal distribution and homogeneity of variance was conducted by paired *t* test, while non‐paired design was analysed by non‐paired *t* test. Comparison between multiple groups was conducted by one‐way analysis of variance, followed by Tukey's post hoc test. Data comparison between multiple groups at different time‐points was conducted by repeated‐measures analysis of variance, followed by Bonferroni correction. A value of *P* < .05 was considered to be statistically significant.

## 3 RESULTS

### 3.1 LINC00176 is highly expressed in ovarian cancer

**Figure 1:**
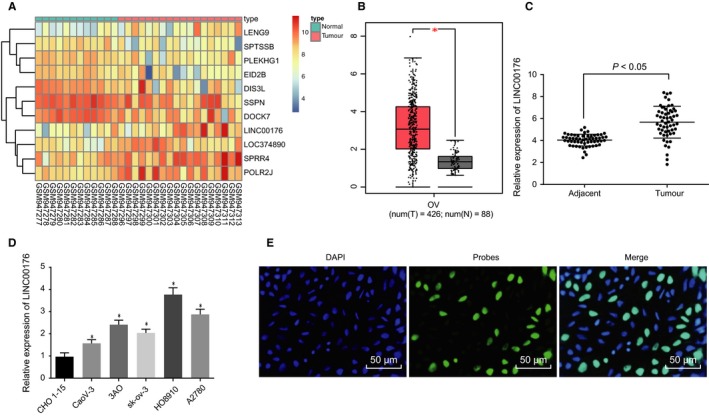
High expression of LINC00176 is observed in ovarian cancer tissues and cells. A, Heatmap illustrating differentially expressed genes related to ovarian cancer, plotted by sample number on the horizontal axis and differentially expressed genes on the vertical; histogram represents colour gradation, and each rectangle represents expression value of each sample. B, Expression of LINC00176 in GEPIA database. C, Expression of LINC00176 in ovarian cancer tissues (n = 56) and adjacent tissues (n = 23) determined by RT‐qPCR. D, Expression of LINC00176 in 5 ovarian cancer cell lines (CaoV‐3, 3AO, sk‐ov‐3, HO8910 and A2780) and normal ovarian epithelium cell line CHO 1‐15 determined by RT‐qPCR; *P < .05 vs the CHO 1‐15 cell line. E, Subcellular localization of LINC00176 in HO8910 cells detected by FISH assay (200×). All data were measurement data and expressed as mean ± standard deviation. Comparison between two groups was analysed by paired *t* test. Comparison among multiple groups was conducted by one‐way analysis of variance, followed by Tukey's post hoc test. Each reaction was run in triplicate

Initially, the ovarian cancer‐related microarray data in combination with Gene Expression Profiling Interactive Analysis (GEPIA) (http://gepia.cancer-pku.cn/index.html) were performed to identify the respective differentially expressed lncRNAs, the results of which demonstrated LINC00176 as a significantly highly expressed lncRNA in ovarian cancer (Figure 1A and B). Besides, the expression profile of LINC00176 in 56 pairs of ovarian cancer and adjacent tissues was explored by conducting RT‐qPCR and the results showed a higher expression profile of LINC00176 in ovarian cancer tissues compared to the adjacent tissues (*P* < .05, Figure 1C). Then, an investigation was conducted to explore the expression pattern of LINC00176 in 5 ovarian cancer cell lines (CaoV‐3, 3AO, sk‐ov‐3, HO8910 and A2780) and normal ovarian epithelium cell line CHO 1‐15 (ATCC, CRL‐9606) (Figure 1D). A significantly increased expression of LINC00176 was found in all 5 ovarian cancer cell lines (*P* < .05), of which the HO8910 cell line exhibited the highest LINC00176 expression profile and therefore was selected as the subject for the following experiments. Subcellular localization of LINC00176 in HO8910 cells detected using FISH assay (Figure 1E) demonstrated that LINC00176 was evenly distributed in the nucleus, indicating that LINC00176 was primarily localized in the nucleus. Taken together, these findings suggested that LINC00176 was expressed at a high level in ovarian cancer.

### 3.2 Silencing LINC0176 impedes EMT of ovarian cancer cells

**Figure 2:**
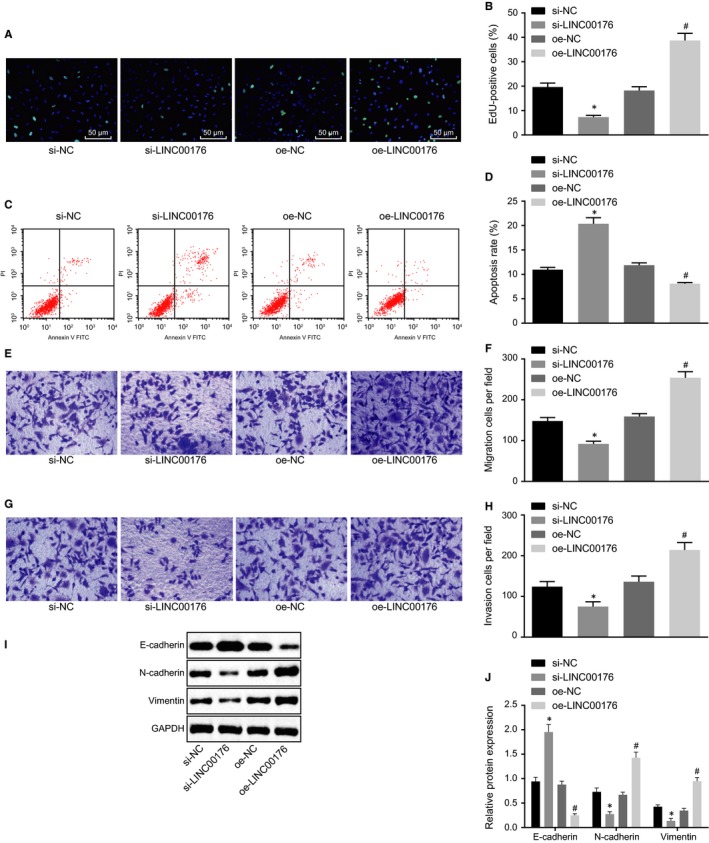
Silencing LINC0176 exerts inhibitory effects on EMT of ovarian cancer cells. A and B, Cell proliferation detected by EdU assay (200×). C and D, Cell apoptosis measured by flow cytometry. E and F, Cell migration detected by Transwell assay (200×). G and H, Cell invasion detected by Transwell assay (200×). I and J, Western blot analysis of EMT‐related proteins (E‐cadherin, N‐cadherin and vimentin) normalized to GAPDH. *P < .05 vs the si‐NC group (HO8910 cells treated with si‐NC); #*P* < .05 vs the oe‐NC group (HO8910 cells treated with oe‐NC). All data were measurement data and expressed as mean ± standard deviation. Comparison between two groups was analysed by non‐paired *t* test. Each reaction was run in triplicate

After identification of the high expression of LINC00176 in ovarian cancer, the aim was shifted onto assessing the effects of LINC00176 on different phenotypes of ovarian cancer cells following transduction of si‐LINC00176 and oe‐LINC00176. In comparison with the cells treated with si‐NC, treatment with si‐LINC00176 led to the suppression of cellular proliferation (Figure 2A and B), migration and invasion (Figure 2E‐H) but it also promoted apoptosis (Figure 2C and D). Contradictory changing tendency was observed in the presence of oe‐LINC00176 compared to oe‐NC (Figure 2A‐H). EMT of ovarian cancer cells was also depicted by quantification of EMT‐related factors (E‐cadherin, N‐cadherin and vimentin) after LINC00176 expression was depleted by delivery of si‐LINC00176 or restored by oe‐LINC00176, respectively. Results showed that si‐LINC00176 resulted in a higher protein level of E‐cadherin but lower protein levels of N‐cadherin and vimentin, while oe‐LINC00176 led to opposite results (*P* < .05, Figure 2I and J). The aforementioned findings demonstrated that silencing LINC00176 to be sufficient to weaken the ovarian cancer cell capabilities of proliferation, migration and invasion as well as to enhance the apoptotic capability, leading to suppressed EMT.

### 3.3 Overexpressed LINC00176 up‐regulates expression of CP *via* BCL3

**Figure 3:**
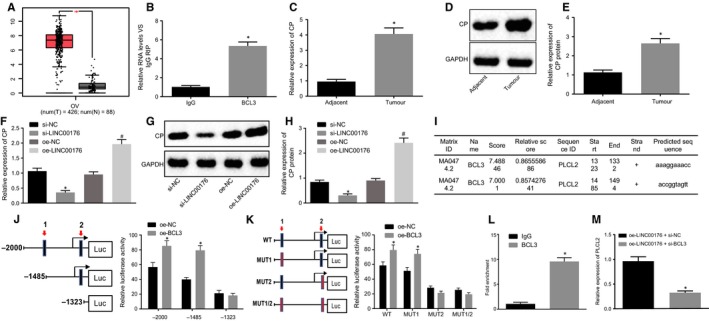
LINC00176 promotes CP expression by recruiting BCL3. A, Expression of CP in ovarian cancer according to GEPIA database. B, Binding between LINC00176 and BCL3 detected by RIP assay; *P < .05 vs the IgG group. C, mRNA expression of CP in ovarian cancer and adjacent tissues determined by RT‐qPCR; *P < .05 vs the adjacent tissues. D and E, Western blot analysis CP protein in ovarian cancer and adjacent tissues normalized to GAPDH; *P < .05 vs the adjacent tissues. F, mRNA expression of CP in HO8910 cells after transfection determined by RT‐qPCR; *P < .05 vs the si‐NC group (HO8910 cells treated with si‐NC); #*P* < .05 vs the oe‐NC group (HO8910 cells treated with oe‐NC). G and H, Western blot analysis of CP protein in HO8910 cells normalized to GAPDH; *P < .05 vs the si‐NC group (HO8910 cells treated with si‐NC); #*P* < .05 vs the oe‐NC group (HO8910 cells treated with oe‐NC). I, Two possible binding sites between BCL3 and CP DNA predicted online. J and K, Binding between BCL3 and CP DNA verified by dual‐luciferase reporter gene assay; *P < .05 vs the si‐NC group (HO8910 cells treated with si‐NC) or oe‐NC group (HO8910 cells treated with oe‐NC). L, Binding between BCL3 and CP DNA on site 2 detected by ChIP assay; *P < .05 vs the IgG group. M, CP expression in HO8910 cells after transfection of oe‐LINC00176 + si‐NC and oe‐LINC00176 + si‐BCL3 determined by RT‐qPCR; * *P* < .05 vs the oe‐LINC00176 + si‐NC group (HO8910 cells treated with oe‐LINC00176 + si‐NC). All data were measurement data and expressed as mean ± standard deviation. Comparison between two groups was analysed by non‐paired *t* test. Each reaction was run in triplicate

Subsequently, an investigation was conducted to explore the molecular mechanism of LINC00176 in ovarian cancer. According to the LncMAP website (http://bio-bigdata.hrbmu.edu.cn/LncMAP/), LINC00176 regulated CP expression by BCL3, and the GEPIA database revealed that CP was highly expressed in ovarian cancer (Figure 3A). The results obtained from RIP assay presented in Figure 3B showed that BCL3 was verified to specifically bind to LINC00176 as evidenced by significantly more LINC00176 enrichment caused by BCL3 than IgG (*P* < .05). Then, RT‐qPCR and Western blot analysis were conducted to quantify CP expression in ovarian cancer and adjacent tissues, the results of which showed that CP was abnormally overexpressed in ovarian cancer as the mRNA and protein expression of CP was much higher in ovarian cancer tissues than adjacent tissues (*P* < .05, Figure 3C‐E). To further explore the regulatory role of LINC00176 in CP, HO8910 cells were treated with oe‐LINC00176 or si‐LINC00176, followed by quantification of the CP expression using RT‐qPCR and Western blot analysis. Results revealed that oe‐LINC00176 treatment significantly elevated the mRNA and protein expression of CP while si‐LINC00176 resulted in significantly reduced mRNA and protein expression of CP (*P* < .05, Figure 3F‐H). Therefore, it could be concluded that restoration of LINC00176 up‐regulated CP expression.

Next, the study focused on conceding the underlying mechanism between LINC00176 and CP. Analysis results from UCSC (http://genome.ucsc.edu/) and JASPAR (http://jaspar.genereg.net/) revealed the presence of 2 binding sites between BCL3 and CP DNA (Figure 3I). Dual‐luciferase reporter gene assay was introduced for verification purposes and the results demonstrated that site 2 was crucial to the relative luciferase activity of CP as the capability of oe‐BCL3 activating CP was significantly weakened with the truncated or mutant site 2 (*P* < .05), while no significant difference was observed with the truncated or mutant site 1 (*P* > .05, Figure 3J and K), thereby suggesting site 2 as the binding site between BCL3 and CP promoter. Moreover, the binding ability of BCL3 to CP DNA on site 2 was detected by ChIP assay, the results of which showed that relative enrichment of CP DNA by BCL3 significantly increased compared to IgG (*P* > .05, Figure 3L), ascertaining site 2 of CP DNA as the binding site to BCL3. The aforementioned findings elucidated that LINC00176 could promote the transcription expression of CP by recruiting BCL3.

In order to further explore the regulatory mechanism among LINC00176, BCL3 and CP, rescue experiments were designed with transfection of oe‐LINC00176 + si‐NC and oe‐LINC00176 + si‐BCL3, followed by RT‐qPCR for quantification of CP expression (Figure 3M). Results showed that transfection of oe‐LINC00176 + si‐BCL3 significantly diminished CP expression while transfection of oe‐LINC00176 + si‐NC did not, suggesting that silencing BCL3 impeded the promoting effect of LINC00176 on CP expression. The above‐mentioned findings offered evidence proving that LINC00176 promoted CP expression in ovarian cancer through recruitment of BCL3.

### 3.4 LINC00176/BCL3 involves in the regulation of EMT in ovarian cancer

**Figure 4:**
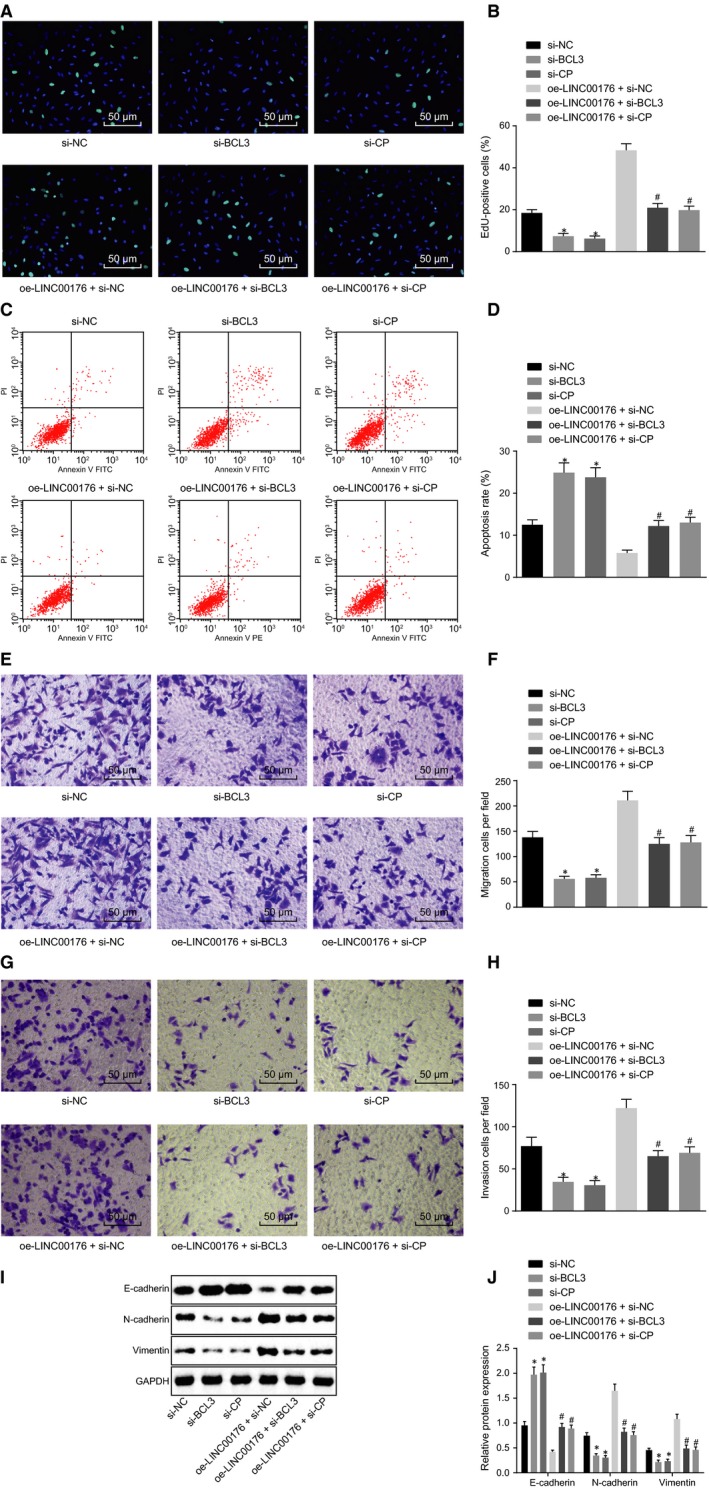
BCL3 and CP participate in the regulation of EMT of ovarian cancer cells by LINC00176. A and B, Cell proliferation detected by EdU assay (200×). C and D, Cell apoptosis measured by flow cytometry. E and F, Cell migration detected by Transwell assay (200×). G and H, Cell invasion detected by Transwell assay (200×). I and J, Western blot analysis of EMT‐related proteins (E‐cadherin, N‐cadherin and vimentin) normalized to GAPDH. *P < .05 vs the si‐NC group (HO8910 cells treated with si‐NC); #*P* < .05 vs the oe‐LINC00176 + si‐NC group (HO8910 cells treated with oe‐LINC00176 + si‐NC). All data were measurement data and expressed as mean ± standard deviation. Comparison among multiple groups was conducted by one‐way analysis of variance, followed by Tukey's post hoc test. Each reaction was run in triplicate

With results determining the functionality of the regulatory mechanism regarding LINC00176, BCL3 and CP, effects induced by such mechanisms in ovarian cancer were then investigated with regard to cell proliferation, apoptosis, migration, invasion and EMT after transduction of si‐BCL3, si‐CP and oe‐NICL00176. It was observed that both si‐BCL3 and si‐CP exerted inhibitory effects on cell proliferation (Figure 4A and B), migration (Figure 4E and F) and invasion (Figure 4G and H) accompanied by accelerated apoptosis (Figure 4C and D), increased E‐cadherin protein level and decreased protein levels of N‐cadherin and vimentin (Figure 4I and J). Results of cells in the presence of oe‐LINC00176 + si‐BCL3 or oe‐LINC00176 + si‐CP were consistent compared to the cells in the presence of oe‐LINC00176 + si‐NC. These findings highlighted overexpressed LINC00176 as a contributing factor to EMT in ovarian cancer while silencing BCL3 or CP can reverse such alternation, which indicated that LINC00176 regulated EMT in ovarian cancer mediated by regulation of the BCL3 and CP protein.

### 3.5 LINC00176 regulates CP expression *via* promotion of the interaction between BCL3 and NF‐κB1 P50

**Figure 5:**
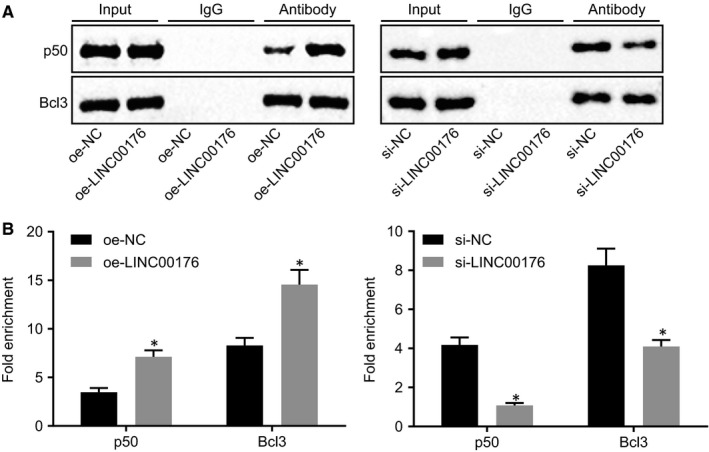
LINC00176 regulates CP expression by promoting the interaction between BCL3 and NF‐κB1 P50. A, The binding of BCL3 to P50 upon oe‐LINC00176 or si‐LINC00176 treatment evaluated using Co‐IP. B, The binding of BCL3 to P50 in the CP promoter and fold enrichment of CP promoter upon oe‐LINC00176 or si‐LINC00176 treatment detected using ChIP assay an RT‐qPCR, respectively. All data were measurement data and expressed as mean ± standard deviation. The diagram on the left indicates oe‐LINC00176 treatment, *P < .05 vs the oe‐NC group (HO8910 cells treated with oe‐NC). The diagram on the right indicates si‐LINC00176 treatment, *P < .05 vs the si‐NC group (HO8910 cells treated with si‐NC). Each reaction was run in triplicate

A former study indicated that BCL3 could closely bind to NF‐κB P50 or p52 homodimer and trans‐activated.^14^ In our study, we initially conducted the Co‐IP experiment and found that BCL3 and P50 bound and interact with each other. Overexpression of LINC00176 promoted the binding of BCL3 to P50, while silencing of LINC00176 impeded the binding of BCL3 to P50 (Figure 5A). Further results of ChIP revealed that overexpressed LINC00176 could promote the binding of BCL3 to P50 in the CP promoter, while silenced LINC00176 could inhibit the binding of BCL3 to P50 in the CP promoter (Figure 5B). These results suggested that LINC00176 stimulated the interaction between BCL3 and NF‐κB1 P50, and then regulated the expression of CP by recruiting the BCL3‐P50 complex in CP promoter.

### 3.6 LINC00176 promotes the development of ovarian cancer in vivo

**Figure 6:**
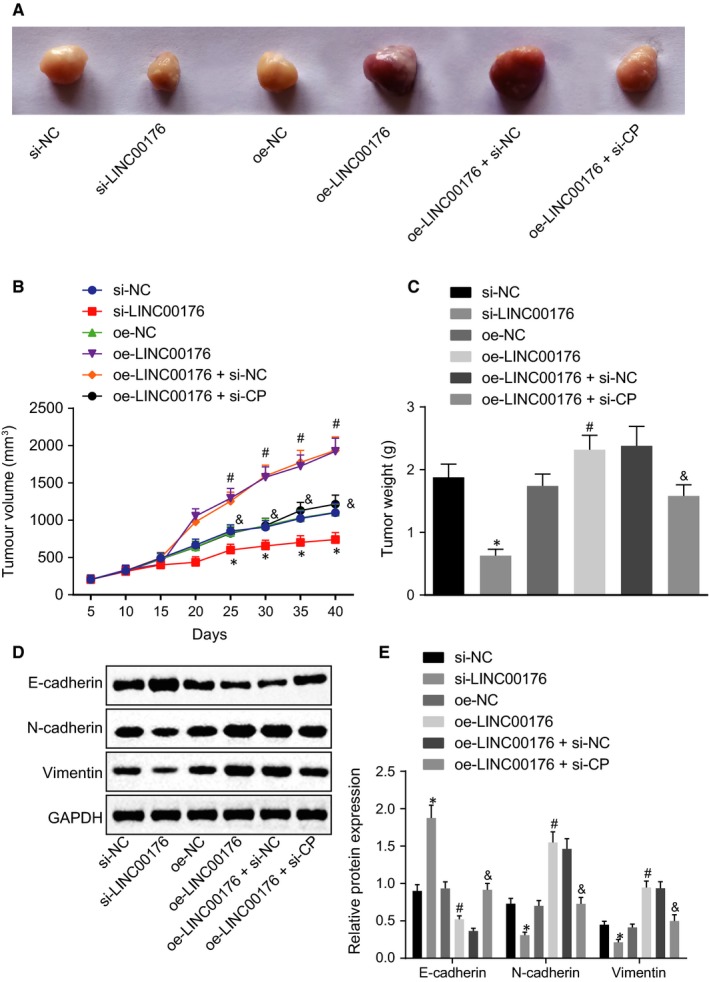
Tumour growth is promoted by LINC00176 restoration which could be reversed by CP silencing in vivo. A, Images of subcutaneous transplanted tumours in nude mice. B, Tumour volume of subcutaneous transplanted tumours in nude mice. C, Statistical analysis of final tumour weight. D and E, Western blot analysis of EMT‐related proteins (E‐cadherin, N‐cadherin and vimentin) normalized to GAPDH. *P < .05 vs the si‐NC group (nude mice bearing HO8910 cells treated with si‐NC); #*P* < .05 vs the oe‐NC group (nude mice bearing HO8910 cells treated with oe‐NC); &*P* < .05 vs the oe‐LINC00176 + si‐NC group (nude mice bearing HO8910 cells treated with oe‐LINC00176 + si‐NC). All data were measurement data and expressed as mean ± standard deviation. Data comparison among multiple groups at different time‐points was conducted by repeated‐measures analysis of variance, followed by Bonferroni correction. Comparison among multiple groups was conducted by one‐way analysis of variance, followed by Tukey's post hoc test. n = 6

Lastly, an in vivo animal model was established to investigate the influence of LINC00176 in ovarian cancer. Mice injected with si‐LINC00176‐treated cells or oe‐LINC00176 + si‐CP‐treated cells exhibited significantly smaller tumour volume and lighter tumour weight in comparison with the injection of cells treated with si‐NC or oe‐LINC00176 + si‐NC (*P* < .05), while mice treated with oe‐LINC00176 had larger tumour volume and heavier tumour weight in comparison with the oe‐NC group (*P* < .05, Figure 6A‐C). Meanwhile, Western blot analysis was conducted to determine the levels of EMT‐related proteins (E‐cadherin, N‐cadherin and vimentin). Higher E‐cadherin level and lower levels of N‐cadherin and vimentin were observed following injection of cells treated with si‐LINC00176 and oe‐LINC00176 + si‐CP, while opposite results were found in mice injected with cells in the presence of oe‐LINC00176 only (*P* < .05, Figure 6D and E). In conclusion, the positive effect of overexpressed LINC00176 on oncogenicity in ovarian cancer was validated successfully in vivo.

## 4 DISCUSSION

According to *Global Cancer Statistics 2018*, 295 414 cases of ovarian cancer have been reported in 2018 worldwide with a mortality rate of approximately 62.56% (184 799/295 414).^15^ Great importance should be attached to the prevention and early detection of ovarian cancer. Promisingly, the prognostic value of lncRNAs in ovarian cancer has been documented, which are mostly up‐regulated.^16^ In the current study, an investigation was conducted to explore the specific role of LINC00176 in ovarian cancer with the underlying regulatory mechanism discovered. Gathered evidence indicates that silencing LINC00176 could potentially inhibit ovarian cancer cell proliferation, migration and invasion while inducing apoptosis by down‐regulating CP *via* recruiting the BCL3 protein, the mechanism of which provides a novel understanding of ovarian cancer progression.

An initial conclusion was comprehended as that LINC00176 was highly expressed in ovarian cancer tissues and cells featured with strengthened cell capabilities of proliferation, migration, invasion and EMT along with weakened apoptotic property. Hitherto, limited studies have enclosed the role of LINC00176 in malignancy. A contrary expression pattern of LINC00176 has been detected in oesophageal cancer and patients exhibiting a higher LINC00176 expression are expected to have a longer overall survival.^9^ In consistency with our results, LINC00176 expression has been found to be expressed at high levels in hepatocellular carcinoma while depleted LINC00176 can induce necroptosis.^8^ To assess the involvement of LINC00176 in the biological behaviours of ovarian cancer cells, we treated HO8910 cells with si‐LINC00176. Consequently, depletion of LINC00176 in HO8910 cells significantly induced cell apoptosis and inhibited cell proliferation, migration and invasion. EMT is the initial process characterized by the metastasis of tumour cells.^17^ The conversion of epithelial cells to mesenchymal cells is vital for the invasion and metastasis of ovarian cancer while the switch from E‐cadherin to N‐cadherin plays a crucial role illustrated by decreased E‐cadherin and increased N‐cadherin.^18^ E‐cadherin, N‐cadherin and vimentin have been identified as phenotype markers for EMT.^19^ Vimentin is ubiquitous in normal mesenchymal cells by functioning as a critical regulator of cellular integrity and overexpression of vimentin is indicative of enhanced tumour growth, accelerated invasion and dismal prognosis.^20^ We identified EMT to be suppressed by silencing LINC00176 as evidenced by higher E‐cadherin level and lower levels of N‐cadherin and vimentin. Likewise, overexpressed lncRNA SPRY4 intronic transcript 1 has been demonstrated to up‐regulate E‐cadherin and down‐regulate N‐cadherin and vimentin, which would consequently impede metastasis through EMT in ovarian cancer.^21^ Furthermore, in other types of cancers, EMT is closely increased by many lncRNAs such as TRE, MALTA1 and HOTAIR.^22^ Therefore, it could be speculated that lncRNAs usually participate in the EMT of tumorigenesis.

Finally, it is well acknowledged that lncRNA recruiting a variety of transcription factors is a common pattern that regulates viability and metastasis of tumour cells.^23^ Transcription factors have been elucidated to mediate gene expression and are involved in the regulation of proliferative status, cellular differentiation and cell fate.^24^ LncRNAs usually recruit these proteins forming RNP complex and specifically binding to the promoter region of target genes. For instance, the regulatory role of lncRNA metastasis‐associated lung adenocarcinoma transcript 1 (MALAT1) in cell cycle progression is facilitated *via* the oncogenic transcription factor B‐MYB.^25^ Currently, no study has reported the relation between BCL3 and lncRNAs. In our study, a novel discovery revealed that LINC00176 could positively regulate the expression of CP through regulation of the transcription factor BCL3. BCL3 is known as a transcription factor associated with the activity of NF‐κB concerning the inflammatory response in pancreatic and biliary tissues.^26^ However, future researches are required regarding the regulatory possibility of the NF‐κB signalling pathway in ovarian cancer. Critically, the proto‐oncogene BCL3 has been detected to express highly in human ovarian cancer tissues where ovarian cancer cell survival, proliferation and migration are promoted,^12^ supporting our results with regard to the rescue properties of silencing BCL3.

**Figure 7:**
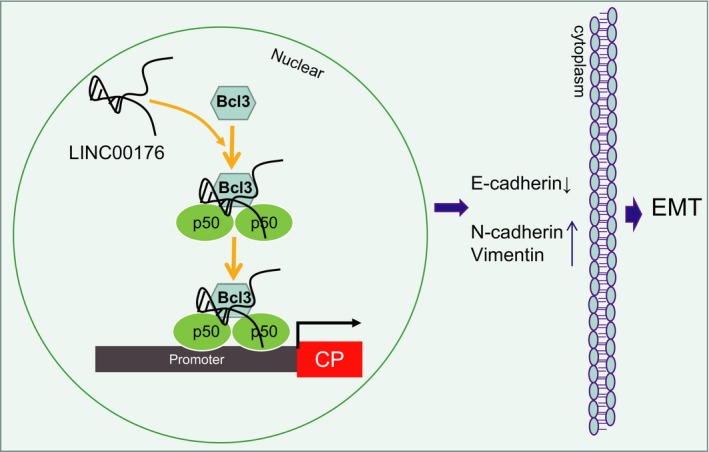
Schematic illustration depicting molecular basis of LINC00176/BCL3/CP regulating EMT of ovarian cancer cells. To be specific, LINC00176 up‐regulates CP expression by recruiting transcription factor BCL3, by which EMT of ovarian cancer cells can be promoted

To conclude, LINC00176 functions as an oncogene, and the reduction in its expression is associated with suppressed tumorigenicity and EMT through inhibition of CP *via* BCL3 (Figure 7). However, it is uncertain that such effects may be induced by different downstream signal pathways like the NF‐κB signalling pathway. Therefore, their exact functions require further confirmation in future studies. The present study can serve as an insight for identifying these factors in early diagnosis or providing a new approach for the treatment of ovarian cancer.

## CONFLICT OF INTEREST

We declare that we have no conflicts of interest.

## AUTHOR CONTRIBUTIONS

Lan Dai and Jixiang Niu designed the study. Yanli Feng collated the data. Lan Dai and Jixiang Niu carried out data analyses and produced the initial draft of the manuscript. Yanli Feng, Lan Dai and Jixiang Niu contributed to drafting the manuscript. All authors have read and approved the final submitted manuscript.

## ETHICAL APPROVAL

The study was conducted under the approval of the Institutional Review Board of Chinese Medicine Hospital in Linyi City. All participants provided written informed consent prior to this study. The animal protocol and experiment procedure were conducted under the approval of the Institutional Animal Care and Use Committee of Chinese Medicine Hospital in Linyi City. Animals in our study were used for research purpose only.

## Data Availability

Not applicable.
